# Assessment of the Prevalence and Drug Susceptibility of *Listeria monocytogenes* Strains Isolated from Various Types of Meat

**DOI:** 10.3390/foods9091293

**Published:** 2020-09-14

**Authors:** Krzysztof Skowron, Ewa Wałecka-Zacharska, Natalia Wiktorczyk-Kapischke, Karolina Jadwiga Skowron, Katarzyna Grudlewska-Buda, Justyna Bauza-Kaszewska, Zuzanna Bernaciak, Miłosz Borkowski, Eugenia Gospodarek-Komkowska

**Affiliations:** 1 Department of Microbiology, Nicolaus Copernicus University in Toruń, Ludwik Rydygier Collegium Medicum, 9 M. Skłodowskiej-Curie Street, 85-094 Bydgoszcz, Poland; natalia12127@gmail.com (N.W.-K.); katinkag@gazeta.pl (K.G.-B.); zuza.bernaciak@gmail.com (Z.B.); milos.borkowski@gmail.com (M.B.); gospodareke@cm.umk.pl (E.G.-K.); 2 Department of Food Hygiene and Consumer Health, Wrocław University of Environmental and Life Sciences, 31 C.K. Norwida St., 50-375 Wrocław, Poland; ewa.walecka@upwr.edu.pl; 3 Institute of Telecommunications and Computer Science, UTP University of Science and Technology, Al. Prof. S. Kaliskiego 7, 85-796 Bydgoszcz, Poland; kj.skowron@wp.pl; 4 Department of Microbiology and Food Technology, UTP University of Science and Technology, 6 Bernardyńska St., 85-029 Bydgoszcz, Poland; justynabauza@gazeta.pl

**Keywords:** *Listeria monocytogenes*, meat, listeriosis, drug susceptibility, antibiotics

## Abstract

*Listeria monocytogenes* are the etiological factor of listeriosis, and their main source for humans is food. The aim of the current study was to assess the contamination of various types of meat and the drug susceptibility of isolated *L. monocytogenes*. Between 2016–2018, 6000 swabs were taken (2000 annually) from the surface of pork, beef, and poultry. The analysis of intermediate and finished product samples was carried out in accordance with ISO 11290-1 (International Organization for Standardization). The genetic similarity assessment of the isolates obtained was based on the Pulsed Field Gel Electrophoresis (PFGE) method, and drug-sensitivity assessment using the disc-diffusion method. We found 2.1% of collected samples were *L. monocytogenes* positive. The level of meat contamination varied depending on its matrix. Most *L. monocytogenes* were isolated from poultry. It was shown that 39 (32.5%) strains were sensitive to all tested antibiotics and eight (6.7%) were resistant to all five tested antimicrobials. Most strains tested were resistant to cotrimoxazole (55; 45.8%) and meropenem (52; 43.3%), followed by erythromycin (48; 40.0%), penicillin (31; 25.8%), and ampicillin (21; 17.5%). High prevalence of this pathogen may be a serious problem, especially when linked with antibiotic resistance and high percentage of serotypes responsible for listeriosis outbreaks.

## 1. Introduction

*Listeria monocytogenes* are Gram-positive, rod-shaped, facultatively anaerobic bacterium. It was demonstrated that *L.* *monocytogenes* can be isolated from various environments including soil, vegetation, surface water, sewage, animal feeds, farm environments, and food-processing environments. *L. monocytogenes* are the etiological factor of human listeriosis. People particularly vulnerable to infection are the elderly, pregnant women, newborns, and immunocompromised individuals. In adult males and non-pregnant women listeriosis causes primary meningitis, encephalitis or septicaemia [1,2,3,4]. Due to the high mortality rate (15.6%) listeriosis is considered one of the most serious foodborne zoonoses in the European Union [5].

A wide range of foodstuffs are reported as the potential source of *L. monocytogenes*—soft cheese, sausage, unpasteurized milk and dairy products, meats, smoked fish, vegetables, salads, and ready-to-eat (RTE) products [3,4,6,7,8]. On the basis of the results of the Rapid Alert System for Food and Feed (RASFF) investigation, it can be stated that the products of animal origin are mostly linked to *L. monocytogenes* infections [9]. Raw poultry and meats, processed meat, and RTE meat products can be contaminated with *L. monocytogenes* in the slaughterhouse or on different processing steps [6,10,11,12,13]. It is suggested that most of the meat processing plants may be colonized by *L. monocytogenes* as soon as during initial production cycles as an effect of raw meat contamination or improper hygienic conditions in the food processing environment (FPE) [1,14]. According to European Food Safety Authority (EFSA), *L*. *monocytogenes* was detected in 1.3%, 0.6%, and 3.1% of pig, poultry, and bovine meat product RTE samples in 2018, respectively [5].

According to the RASFF report, three of six multi-country foodborne outbreaks reported in Europe in 2018 were related to *L. monocytogenes* and all of them have led to a Rapid Outbreak Assessment (ROA) [9]. In the largest *L. monocytogenes* outbreak caused by RTE meat products in South Africa in 2017–2018, more than 1000 hospitalizations and 200 deaths were reported [15,16,17]. The causative agent was the *L. monocytogenes* strain of serogroup 4b, multi-locus sequence type 6 (ST6). The same serotype was identified as a cause of the multi-country (Austria, Denmark, Finland, Sweden, UK) *L. monocytogenes* outbreak linked to frozen vegetables (corn) between 2015–2018, with 47 confirmed listeriosis cases and nine lethal cases [18].

Since *L. monocytogenes* pathogenicity and persistence in FPE may be related to its serotype, the serological identification of *L. monocytogenes* strain isolated from food is of major importance for consumer safety [1]. About 90–95% of the strains isolated from contaminated food, animal, and human samples belong to serotypes 1/2a, 1/2b, 1/2c, and 4b, among which mentioned serotype 4b is responsible for the majority of human listeriosis cases and 1/2a serotype is the most prevalent in foods [1,4,6,7,10,17,19].

The risk of *L. monocytogenes* occurrence in FPE may increase due to the drug resistance of pathogenic strains. Although *L. monocytogenes* is thought to maintain a relatively high susceptibility to most of the clinically relevant antimicrobials, the anthropogenic activity (extensive use of antibiotics, cross-resistance to molecules used as growth promoters in animal production) can result in spreading antibiotic resistance among *L. monocytogenes* strains [4,20].

The aim of the current study was to assess the prevalence of *Listeria monocytogenes* in various types of meat (pork, beef, and poultry) between 2016–2018. Identification of the serotypes and characteristics of their profile of resistance to antibiotics were also investigated.

## 2. Materials and Methods

### 2.1. Material

The research material included 6000 swabs taken from the surface of pork, beef, and poultry in 2016–2018. Every year, 2000 samples were taken, respectively: (1) 610 from poultry, 685 from pork, and 705 from beef in 2016; (2) 650 from poultry, 715 from pork, and 635 from beef in 2017; and (3) 690 from poultry, 684 from pork, and 626 from beef in 2018. All samples of a given meat-type were collected from the same meat plant.

Samples for testing were collected by wet swab method with the use of a sterile, flexible template limiting the swabbed area to 100 cm^2^. Samples were taken for the part of the carcass currently processed in the plant (one sample per carcass part). A new sterile template was used for each part of the carcass. A sterile 50 cm^2^ cellulose sponge (Environscreen, Technical Service Consultants Ltd., Heywood, UK) soaked in 10 mL 0.9% NaCl sterile packed in a reinforced zip-bag was used for sampling.

### 2.2. Isolation of L. monocytogenes Strains

Analysis of the meat samples was based on the procedures of the PN-EN ISO 11290-1:1999/A1:2005 [21]. The sponges taken from meat samples were immersed in 100 mL of half-Fraser broth (Merck, Darmstadt, Germany) and incubated at 30 °C for 24 h. The sampling sponge was then squeezed firmly several times into the bag. Secondary selective enrichment was performed for 48 h at 37 °C after transferring 0.1 mL of the culture into 9.9 mL of Fraser broth (Merck, Darmstadt, Germany). Next, a reductive inoculation of the bacterial cultures was performed on the selective agar medium according to Ottaviani and Agosti (ChromoCult^®^ *Listeria* Selective Agar (ALOA^®^; Merck, Darmstadt, Germany). Cultures were incubated for 24 h at 37 °C.

Bacterial colonies suspect to belong to *Listeria* spp. (one colony for each sample) were transferred to Columbia Agar with 5% sheep blood (bioMérieux, Marcy-l’Étoile, France). The hemolysis type was assessed and final identification using the PCR method, was performed. The identified *L. monocytogenes* isolates were frozen in brain-heart infusion broth (BHI; Merck, Darmstadt, Germany) with 15% glycerol (Avantor, Gliwice, Poland) and stored at −80 °C.

### 2.3. Isolation of Genomic DNA

In order to isolate *L. monocytogenes* genomic DNA, the column method with the Genomic Mini AX Bacteria Spin Kit (A&A Biotechnology, Gdynia, Poland) was applied, according to the protocol provided by the manufacturer.

### 2.4. Species Identification

The identification of *L. monocytogenes* isolates was confirmed by PCR reaction. Two pairs of primers L1 (5′-CAG CAG CCG CGG TAA TAC-3′), L2 (5′-CTC CAT AAA GGT GAC CCT-3′), LM1 (5′-CCT AAG ACG CCA ATC GAA-3′) and LM2 (5′-AAG CAC TTG CAA CTG CTC-3′; Oligo.pl) were applied [22]. The PCR reaction mixture (25 μL) included 1× PCR buffer (Promega, Madison, WI, USA), 2 mM MgCl_2_ (ABO, Gdańsk, Poland), 1.25 mmol dNTPs (Promega, Madison, WI, USA), 0.5 μM of each primer (Oligo.pl), 1 unit of Taq DNA polymerase (Promega, Madison, WI, USA), ultrapure water and DNA isolated from *L. monocytogenes*. The PCR program included: initial denaturation 94 °C for 2 min; 30 cycles of denaturation 94 °C for 30 s, annealing 50 °C for 30 s and primer elongation 72 °C for 1 min; extension 72 °C for 5 min.

The PCR products were analyzed by agarose gel electrophoresis (1.5% agarose) stained with Midori Green (NIPPON Genetics EUROPE GmbH, Düren, Germany) in 1× TBE (Tris-boran-EDTA) buffer (BioRad, Hercules, CA, USA) using a DNA size standard (GeneRuler™ 1000 bp DNA Ladder; Fermentas, Waltham, MA, USA)—conditions 90 V, 1 h.

### 2.5. Evaluation of Pulsotypes Similarity (PFGE)

The pulsotypes similarity analysis of the confirmed *L. monocytogenes* strains was performed with the pulsed-field gel electrophoresis (PFGE). The procedure was carried out according to the standard operating procedure for PulseNet PFGE of *L. monocytogenes* [23].

The degree of pulsotypes similarity between analyzed *L. monocytogenes* isolates was evaluated using a phylogenetic dendrogram drawn in the CLIQS 1D Pro program (TotalLab, Newcastle upon Tyne, UK). Clustering analysis was performed using hierarchical clustering with the Unweighted Pair-Group Method Using Arithmetic Averages (UPGMA) technique and Dice’s coefficient.

### 2.6. Molecular Serotyping of L. monocytogenes Strains

In order to identify the main *L. monocytogenes* serogroups (1/2a-3a, 1/2b-3b, 1/2c-3c, 4b-4d-4e) multiplex PCR was applied in accordance to Doumith et al. [24]. For serogroups identification the *L. monocytogenes* reference strains tested by Wałecka-Zacharska et al. [25] were used.

### 2.7. Drug Susceptibility Analysis

The disk-diffusion method was applied to determine antibiotic susceptibility of *L. monocytogenes* strains tested. For this purpose, 24 h bacterial cultures diluted in 0.9% saline solution (Avantor, Gliwice, Poand) were plated on MHF medium (Mueller Hinton Agar with 5.0% horse blood and 20 mg/L β-NAD; bioMérieux, Marcy-l’Étoile, France). Next, following antibiotic discs were added: penicillin (1 IU), ampicillin (2 μg), meropenem (10 μg), erythromycin (15 μg), and cotrimoxazole (1.25–23.75 μg). The antibiotics for the studies were selected in accordance with the European Committee on Antimicrobial Susceptibility Testing (EUCAST) v. 8.0 [26] recommendations. The antibiotics used in the study are the only ones that can be used in Europe to treat listeriosis and for which the interpretation of the results is possible. After 20 h of incubation at 35 °C, growth inhibition zones around the antibiotic discs were measured and analyzed in accordance with the EUCAST v. 8.0 [26].

### 2.8. Statistical Analysis

The obtained results were subjected to statistical analysis in the Statistica 13 PL program (StatSoft, Round Rock, TX, USA). The normality of the distribution of the obtained results was assessed with the Shapiro-Wilko test. ANOVA with the Tukey post-hoc test was used to determine significant differences between strains number in particular groups.

## 3. Results

**Figure 1:**
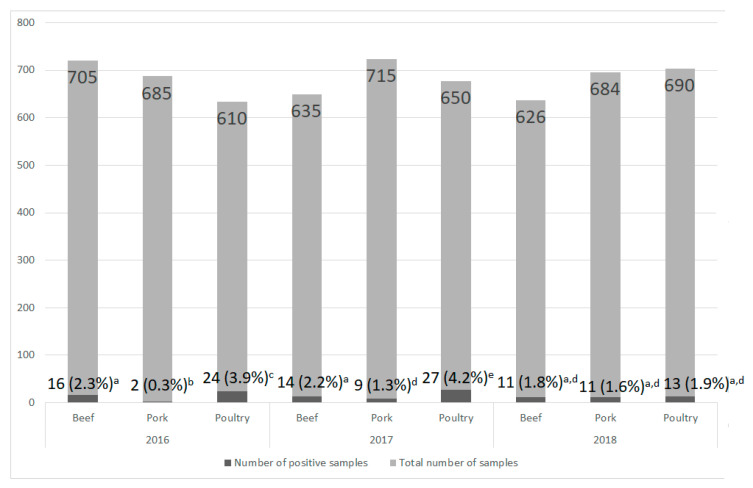
The number of contaminated meat samples depending on the year of analysis and meat type (a, b, c, …—variables marked with at least one different letter differ statistically significantly from the others (*p* < 0.05)).

The research showed that *L. monocytogenes* was isolated from 127 (2.1%) of 6000 swabs taken from all kind of meat. The level of contamination of all meat samples, regardless of their type, in particular years was similar and ranged from 1.8% (35 positive samples) in 2018 to 2.5% (50 positive samples) in 2017. These differences were not statistically significant. In each year, the largest number of positive samples was obtained from poultry (1.9–4.2%), and in 2016–2017 the differences were statistically significant (Figure 1). In turn, the lowest *L. monocytogenes* contamination was noted in pork samples (0.3–1.6%; Figure 1). In 2016–2017, the percentage of contaminated pork samples was statistically significantly lower compared to other types of meat (Figure 1).

### 3.1. Evaluation of Pulsotypes Similarity (PFGE)

**Figure 2:**
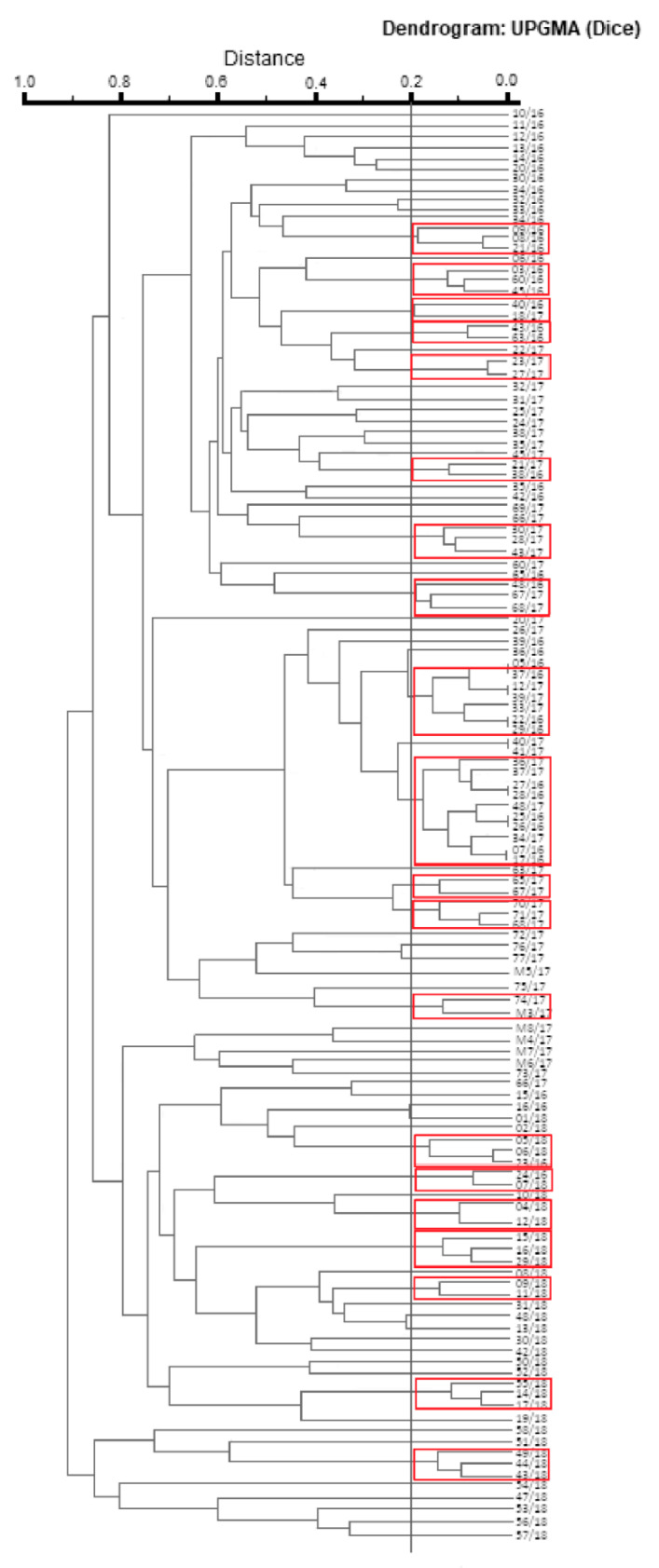
Pulsotypes similarity dendrogram of *L. monocytogenes* isolates tested. The clusters are marked with red rectangles; UPGMA—Unweighted Pair-Group Method Using Arithmetic Averages.

The analysis of pulsotypes similarity (PFGE) distinguished the *L. monocytogenes* isolates into 127 patterns, among which seven pairs of isolates represent the identical pulsotypes (Figure 2). In most cases, strains with identical pulsotypes were obtained from the same material. The exception was a pair of strains 07/16 and 17/16, which were isolated from pork and beef, respectively. Moreover, all strains with identical pulsotypes were always isolated in the same year. The cut-off value was set at 80% similarity. The comparison of pulsotypes allowed us to assign 62 (48.8%) of the tested strains to 20 clusters. Apart from genetically identical strains, the highest degree of similarity (95.0%) was shown by three pairs of isolates: no. 08/16 and 21/16 (isolated in the same year from pork); 23/17 and 27/17 (isolated in the same year from beef and pork, respectively); 06/18 and 23/16 (isolated in 2018 and 2016, respectively from pork). However, the analysis of pulsotypes showed that strains from pairs: 08/16, 21/16 and 06/18, 23/16 were the least similar with strains 09/16 (isolated from beef in 2016) and 05/18 (isolated from pork in 2018), respectively. The remaining 65 (51.2%) strains created unique PFGE patterns and were not classified into any of the clusters at the adopted cut-off value.

### 3.2. Drug Susceptibility Evaluation

**Figure 3:**
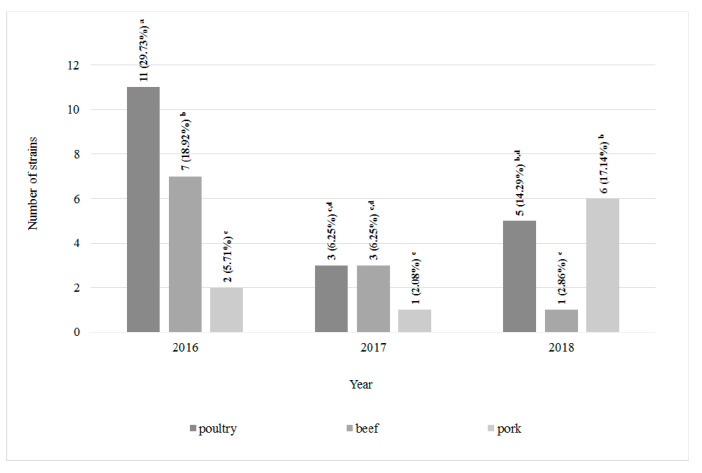
The number of *L. monocytogenes* strains susceptible to all antibiotics tested (*n* = 120; a, b, c, …—variables marked with at least one different letter differ statistically significantly from the others (*p* < 0.05)).

**Figure 4:**
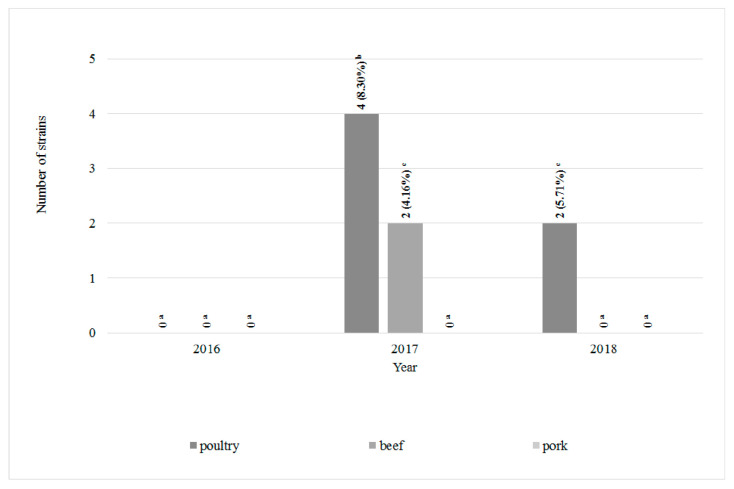
The number of *L. monocytogenes* strains resistant to all antibiotics tested (*n* = 120; a, b, c, …—variables marked with at least one different letter differ statistically significantly from the others (*p* < 0.05)).

Among 120 *L. monocytogenes* strains isolated from all types of meat 22 antimicrobial resistance patterns were defined (Table 1). Profile I included 39 (32.5%) strains susceptible to all antibiotics tested. Statistically the highest number (11, 29.7%) of all antibiotic-susceptible strains was reported in poultry meat in 2016 (Table 1 and Figure 3). On the other hand, eight *L. monocytogenes* strains, classified to profile III, showed resistance to all antibiotics used in the research—six were isolated from poultry and beef meat (four and two, respectively) in 2017 and two from poultry meat in 2018. *L. monocytogenes* strains resistant to all antibiotics has not been isolated from pork (Table 1 and Figure 4). Resistance to cotrimoxazole only (profile II) was confirmed in 14 strains (11.67%). Seven strains (5.8%) were ampicillin-susceptible only (profile IV). Profiles XVII–XXII were represented by single *L. monocytogenes* strains. In 2017 and 2018, the statistically significantly largest number of strains resistant to all tested antibiotics were isolated from poultry (Figure 4).

**Figure 5:**
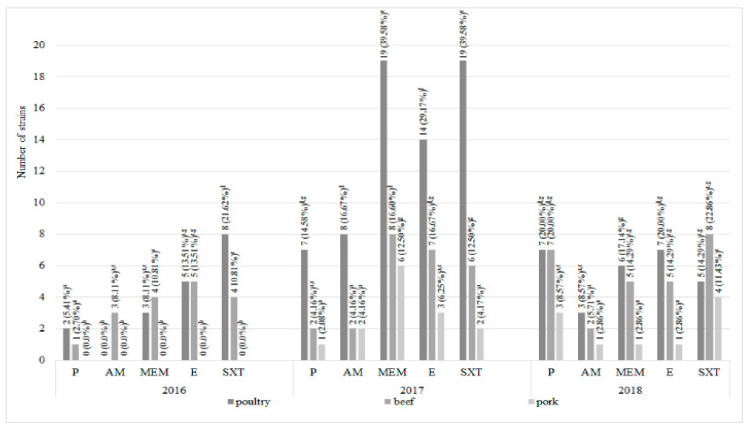
The drug-resistance of *L. monocytogenes* strains tested (*n* = 120; a, b, c, …—variables marked with at least one different letter differ statistically significantly from the others (*p* < 0.05)); P, penicillin; AM, ampicillin; MEM, meropenem; E, erythromycin; SXT, cotrimoxazole.

Overall analysis of *L. monocytogenes* drug susceptibility showed that the most numerous group included strains resistant to cotrimoxazole (55; 45.8%) and meropenem (52; 43.3%). The remaining antimicrobials—erythromycin, penicillin and ampicillin—showed no activity against 48 (40.0%), 31 (25.8%), and 21 (17.5%) strains, respectively (Table 1). The results of the first-year analyses (2016) confirmed the presence of *L. monocytogenes* antibiotic resistant strains in poultry and beef only and 12 of these strains were resistant to cotrimoxazole (32.4%). On the contrary, for the next two years, all the types of meat tested were contaminated with antibiotic resistant strains. Statistically, the highest number of antibiotic-resistant strains was isolated from poultry meat in 2017—19 strains were resistant to cotrimoxazole and the same number to meropenem. It was reported, that the lowest number of *L. monocytogenes* antibiotic resistant strains was isolated from pork meat during each year of the experiment (Figure 5).

### 3.3. L. monocytogenes Serotypes Determination and Distribution in Meat

**Figure 6:**
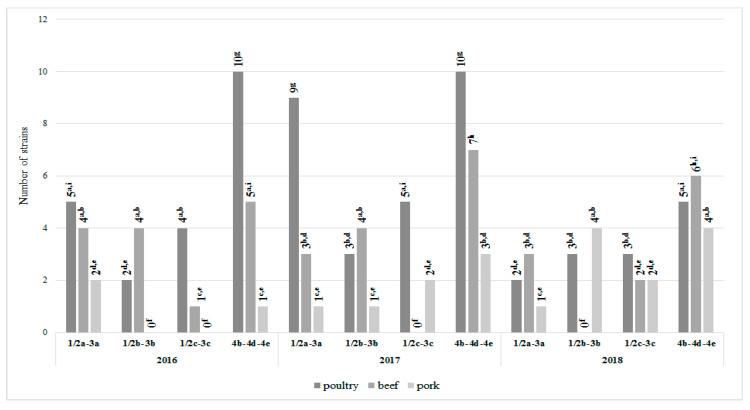
Frequency of *L. monocytogenes* serotypes in meat tested (*n* = 120; a, b, c, …—variables marked with at least one different letter differ statistically significantly from the others (*p* < 0.05)); P, penicillin; AM, ampicillin; MEM, meropenem; E, erythromycin; SXT, cotrimoxazole.

The serotype distribution of analyzed *L. monocytogenes* strains in meat samples was shown in Figure 6. It was found that all four main serogroups were identified within tested *L. monocytogenes* strains.

The most common serogroup isolated from all types of meat samples was 4b-4d-4e (51 strains, 42.5%), followed by 1/2a-3a (29 strains, 24.2%), 1/2b-3b (21 strains, 17.5%), and 1/2c-3c (19 strains, 15.8%). Almost 50% of all 4b-4d-4e strains originated from poultry (Figure 6), it was a statistically significant value. In turn, the least numerous serotype in this type of meat was 1/2b-3b, represented by eight strains only. The 1/2c-3c and 1/2a-3a serogroups were the least frequent within the strains isolated from beef and pork, respectively; it was a statistically significant value (Figure 6).

**Figure 7:**
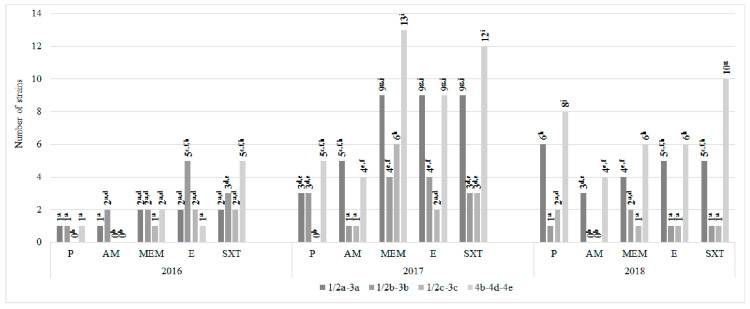
Resistance of *L. monocytogenes* serotypes to antibiotics tested (*n* = 120; a, b, c, …—variables marked with at least one different letter differ statistically significantly from the others (*p* < 0.05)); P, penicillin; AM, ampicillin; MEM, meropenem; E, erythromycin; SXT, cotrimoxazole.

Although the antibiotic resistance of the strains belonging to individual serogroups varied depending on the year of their isolation, some common trends were also observed. Each year of the research the highest number of cotrimoxazole-resistant strains was noted in 4b-4d-4e serotype. Over 50% of strains resistant to cotrimoxazole and meropenem in 2017 (13, 27.1% and 12, 25.0%, respectively) belonged to this serogroup, it was a statistically significant value. A high resistance to all antibiotics tested was confirmed in strains of 1/2a-3a serotype in 2017–2018. On the contrary, isolates of group 1/2c-3c presented a low resistance to penicillin and ampicillin, it was a statistically significant value (Figure 7).

## 4. Discussion

In recent years, the number of listeriosis outbreaks linked to the consumption of contaminated meat food has increased. The presence of *L. monocytogenes* in raw meat may be an effect of various factors, e.g., fecal contamination during evisceration or improper hygiene of employees [27]. Although raw meat is usually cooked before eating, an insufficient thermal processing may not eliminate *L. monocytogenes* from the food, posing a serious health threat for the consumer. Moreover, high incidence of *L. monocytogenes* in raw material significantly increases the risk of secondary contamination of working surfaces and equipment used in the processing plant. In turn, biofilms formed by *L. monocytogenes* on working surfaces may contribute in final product contamination. In our studies the prevalence of *L*. *monocytogenes* in tested meat samples was 2.1% (127/6000). The highest number of isolates was obtained from poultry—64 per 1950 samples (3.3%) followed by beef and pork with 41 per 1966 samples (2.1%) and 22 per 2084 samples (1.1%), respectively. Also, Pesavento et al. [27] observed higher level of *L. monocytogenes* contamination in poultry (39.5%) than in beef (33.3%) and pork (27.3%). The general meta-analysis of data from 21 European countries showed that *L. monocytogenes* was found to be the third most incident pathogen in overall poultry meats [28]. Maung et al. [19] noted that prevalence of *L. monocytogenes* in chicken depended on the year of samples analyses and was 53% and 24%, in 2012 and 2017, respectively. According to Filipello et al. [11] poultry and pork appear to be the second most important source (after dairy products) of human listeriosis cases in Northern Italy. In Gamboa-Marin et al.’s [29] study, pork meat cuts had high *L. monocytogenes* prevalence of 33.9%. On the other hand, Wang et al. [30] found that only 5.5% of chilled pork samples positive for *L. monocytogenes*. Similar to our results, Kuan et al. [31] reported the *L. monocytogenes* prevalence of 33.3% in beef offal from different wet markets. In turn, from the total of 50 beef meat samples examined by Teixiera et al. [4], six were confirmed as *L. monocytogenes* positive (12%). The differences reported in the *L. monocytogenes* prevalence in different meats are affected by various factors, e.g., control measures implementation during the stages of cleaning and disinfection [29].

Antibiotic-resistant (AR) strains of *L. monocytogenes* are serious health risk associated with food consumption. Despite relatively high susceptibility to the antimicrobials commonly used in infection treatment, *L. monocytogenes* has been recently observed to develop increased tolerance to antimicrobials. *L. monocytogenes* antibiotic resistance can result from the acquisition of resistance genes from other Gram-positive bacteria (e.g., plasmid-mediated transfer), however overuse of antimicrobials in medicine and agriculture can also negatively affect the drug susceptibility of the pathogen [20,27,32,33]. Moreover, *L. monocytogenes* cell components and mechanisms involved in the adaptation to stress conditions occurring in the food-chain (the alternative sigma factor B (σ^B^), two-component signal transduction systems, efflux pump activity) were suggested to contribute to the increased pathogen resistance to ampicillin, penicillin, and cephalosporins [32,34]. On the other hand, high sensitivity of *L. monocytogenes* to antimicrobials may be due to the origin of these bacteria in the natural environment where they are not exposed to antibiotics [20].

The results of Maung et al. [19] showed that most *L. monocytogenes* strains isolated from chicken meat displayed antimicrobial susceptibility. Also, Oliveira et al. [35] observed 100% of the isolates were sensitive to most antibiotics tested (except for clindamycin). A high percentage (95%) of strains sensitive to anti-microbials recommended in *Listeria* treatment were also reported by Khen et al. [36]. In Gómez et al.’s [37] research, all *Listeria* strains isolated from ready-to-eat (RTE) meat products and food-processing environments were highly sensitive to the first-choice antibiotics used in listeriosis treatment, such as ampicillin. Similarly, Jorgensen et al. [38] noted sensitivity to ampicillin in all (*n* = 52) *L. monocytogenes* isolates obtained from produce handling and processing (PHP) facilities. In our studies 32.5% of strains (39/120) were susceptible to all antibiotics tested and 82.5% (99/120) displayed sensitivity to ampicillin (Table 1). On the contrary, Carvalho et al. [10] and Sereno et al. [39] detected high level of resistance to ampicillin in *L. monocytogenes* isolated from meat and meat processing environment.

A number of studies confirm antimicrobial resistance of *L. monocytogenes* strains isolated from meat [19,27,36,37,40,41]. In our research 67.5% (81/120) of *L. monocytogenes* strains isolated from meat showed resistance to at least one of the antibiotics tested (Table 1). Barbuti et al. [42] reported resistance to various antibiotics, including erythromycin and cotrimoxazole in meat-originating *L. monocytogenes*. Cotrimoxazole, an antibiotic successfully used to treat infections caused by *L. monocytogenes*, proved to be the least effective against strains isolated in our studies, with (56; 46.7%) isolates resistant (Figure 5). Despite high sensitivity to cotrimoxazole observed in *L. monocytogenes* strains from different steps of a pork production, Sereno et al. [39] found this phenomenon an important concern for public health.

Multi-resistance (multidrug-resistance (MDR); resistance to ≥3 antibiotics) in *L. monocytogenes* strains is currently reported to increase. Maung et al. [19] observed MDR in 46.7% (35/75) and 82.6% (19/23) of *L. monocytogenes* strains isolated from poultry in 2012 and 2017, respectively. Gómez et al. [37] reported multi-resistance of 2.9% of *L. monocytogenes* strains isolated from ready-to-eat (RTE) meat products and food-processing environments. Pesavento et al. [27] found that 51 (30.4%) of pork-originating strains were resistant to three or more antibiotics. Moreover, they observed increasing of percentages *L. monocytogenes* multiresistance. In Wang et al. [30] research 11.5%, 3.85%, and 3.85% *L. monocytogenes* isolated from chilled pork strains were resistant three, six, and nine different antimicrobials, respectively. The results of our analyses showed that 33.3% (40/120) of *L. monocytogenes* from different meat revealed MDR and 6.7% (8/120) were resistant to all antimicrobials (Table 1).

Since *L. monocytogenes* serotypes display various epidemic potential, the analysis of strain serotype may be important in the control and ensuring microbiological food safety [1]. Although there is no conclusive evidence for a correlation between the virulence of *L. monocytogenes* strain and the serotype they represent [43], some serotypes are much more frequently isolated form listeriosis cases than others [44]. Among 14 serotypes determined for *L. monocytogenes* on the basis of 14 somatic (O) and four flagellar (H) antigens, four of them is of special concern for human health and food industry. The most of listeriosis are typically caused by 4b, 1/2b, and 1/2c serotypes and 1/2a serotype is the most common in foods [1,6,45]. It was shown that these serotypes are responsible for over than 95% of the human listeriosis cases [44]. The results of this work agree with those found by other researchers reporting a high frequency of the serotype 4b-4d-4e of *L. monocytogenes* in food industries. Serotype analysis is not very efficient method to differentiate *L. monocytogenes* strains isolated from foods and clinical samples. Because of the genetically heterogeneous nature of *L. monocytogenes*, serotyping as the primary characterization in epidemiological surveillance has been widely accepted [46]. Feng et al. [47] developed a new multiplex PCR assay for rapid detection of new serotype 4h *L. monocytogenes*. These results demonstrate that the multiplex PCR with high specificity and sensitivity is applicable for the rapid detection of *L. monocytogenes* serotypes. Recently, numerous molecular subtyping techniques have been developed for the surveillance or tracing the sources of *L. monocytogenes*, e.g., PFGE [44,48,49].

According to the results of most studies on meat-originating *L. monocytogenes* serotyping suggests the dominance of serotype 1/2a. In Wang et al.’s [30] study, the highest percentage (53.8%) of the *L. monocytogenes* isolated from pork strains belonged to these serogroups, followed by 1/2b (23.1%) and 1/2c (23.1%). Similarly, the presence of these three serogroups within *L. monocytogenes* isolated from chicken meat were reported by Oliveira et al. [35]. The majority of strains belonged to serovar 1/2a (87%), while only 13% to remaining 1/2c and 1/2b. Serotype group 1/2a was also the most commonly isolated (58%) in Khen et al. [36] study on *L. monocytogenes* in bovine hides and meat. Percentage of serogroups 4b, 1/2b, and 1/2c were 12%, 10% and 6%, respectively. Maung et al. [19] conducted research carried out five years apart (2012 and 2017). In 2012 *L. monocytogenes* strains of the four mostly recovered from food, animal and human samples serogroups 1/2a (21.5%), 1/2b (73.9%), 1/2c (1.5%), and 4b/4e (3.1%) were isolated, while in 2017 the presence of only two serotypes 1/2b (51.7%) and 1/2a (48.3%) was confirmed. Contrary to the aforementioned, our results showed that isolates belonging to serotype 4b-4d-4e were the most numerous group 51 (42.5%). Serogroup 1/2a-3a included 29 strains (24.2%), 1/2b-3b 21 strains (17.5%), and 1/2c-3c 19 strains (15.8%; Figure 6). In Teixeira et al.’s [4] study serotype 4 (4b, 4d or 4e) was also the most prevalent among *L. monocytogenes* serogroups. Since the serotype 4b presence in food was linked to serious listeriosis outbreaks, high prevalence in analyzed meat samples should be considered an alarming epidemiological concern [17,50].

The results of the research confirmed *L. monocytogenes* presence in raw poultry, beef, and pork meat. High prevalence of this pathogen may be a serious problem, especially when linked with antibiotic resistance and high percentage of serotypes responsible for listeriosis outbreaks. Although the sensitivity of the *L. monocytogenes* strains tested in our study to penicillin and ampicillin is not so small, the presence of ampicillin-resistant strains of serotype 4b-4d-4e in the analyzed samples is a reason for concern, considering that this serotype is a cause for majority of human listeriosis and ampicillin is a first choice antibiotic in *Listeria*-linked infections treatment. In order to the increase in antibiotic resistance in *L. monocytogenes* emphasized by many studies and researches, the need for constant surveillance of this trend is undisputed.

## Figures and Tables

**Table 1:** Drug susceptibility profiles of the tested *L. monocytogenes* strains (*n* = 120) continuation.

Profile Number	Drug-Resistance Profile	Number												
		2016				2017				2018				Total *n* = 120
		Poultry *n* = 21	Beef *n* = 14	Pork *n* = 2	Total in 2016 *n* = 37	Poultry *n* = 27	Beef *n* = 14	Pork *n* = 7	Total in 2017 *n* = 48	Poultry *n* = 13	Beef *n* = 11	Pork *n* = 11	Total in 2018 *n* = 35	
**I**	R: -	11	7	2	20	3	3	1	7	5	1	6	12	39
	S: P, AM, MEM, E, SXT	(52.4%)	(50.0%)	(100.0%)	(54.1%)	(11.1%)	(21.4%)	(14.3%)	(14.6%)	(38.5%)	(9.0%)	(54.6%)	(32.4%)	(32.5%)
**II**	R: SXT	5	1	0	6	4	0	1	5	0	2	1	3	14
	S: P, AM, MEM, E	(23.8%)	(7.1%)	(0.0%)	(16.2%)	(11.1%)	(0.0%)	(14.3%)	(10.4%)	(0.0%)	(18.2%)	(9.1%)	(8.1%)	(11.7%)
**III**	R: P, AM, MEM, E, SXT,	0	0	0	0	4	2	0	6	2	0	0	2	8
	S: -	(0.0%)	(0.0%)	(0.0%)	(0.0%)	(11.1%)	(14.3%)	(0.0%)	(12.5%)	(15.4%)	(0.0%)	(0.0%)	(5.4%)	(6.7%)
**IV**	R: P, MEM, E, SXT	1	0	0	1	1	0	0	1	2	3	0	5	7
	S: AM	(4.8%)	(0.0%)	(0.0%)	(2.7%)	(3.7%)	(0.0%)	(0.0%)	(2.1%)	(15.4%)	(27.3%)	(0.0%)	(13.5%)	(5.8%)
**V**	R: MEM, E, SXT	1	0	0	1	4	1	0	5	0	0	0	0	6
	S: P, AM	(4.8%)	(0.0%)	(0.0%)	(2.7%)	(11.1%)	(7.1%)	(0.0%)	(10.4%)	(0.0%)	(0.0%)	(0.0%)	(0.0%)	(5.0%)
**VI**	R: MEM	0	1	0	1	2	1	2	5	0	0	0	0	6
	S: P, AM, E, SXT	(0.0%)	(7.1%)	(0.0%)	(2.7%)	(7.4%)	(7.1%)	(28.6%)	(10.4%)	(0.0%)	(0.0%)	(0.0%)	(0.0%)	(5.0%)
**VII**	R: AM, MEM, E, SXT	0	1	0	1	2	0	1	3	0	1	0	1	5
	S: P	(0.0%)	(7.1%)	(0.0%)	(2.7%)	(7.4%)	(0.0%)	(14.3%)	(6.3%)	(0.0%)	(9.1%)	(0.0%)	(2.7%)	(4.2%)
**VIII**	R: MEM, SXT	0	0	0	0	2	2	0	4	0	0	1	1	5
	S: P, AM, E	(0.0%)	(0.0%)	(0.0%)	(0.0%)	(7.4%)	(14.3%)	(0.0%)	(8.3%)	(0.0%)	(0.0%)	(9.1%)	(2.7%)	(4.2%)
**IX**	R: E, SXT	1	2	0	3	1	1	0	2	0	0	0	0	5
	S: P, AM, MEM	(4.8%)	(14.3%)	(0.0%)	(8.1%)	(3.7%)	(7.1%)	(0.0%)	(4.2%)	(0.0%)	(0.0%)	(0.0%)	(0.0%)	(4.2%)
**X**	R: MEM, E	0	0	0	0	1	2	0	3	1	0	0	1	4
	S: P, AM, SXT	(0.0%)	(0.0%)	(0.0%)	(0.0%)	(3.7%)	(14.3%)	(0.0%)	(6.3%)	(7.7%)	(0.0%)	(0.0%)	(2.7%)	(3.3%)
**XI**	R: P	0	0	0	0	0	0	0	0	0	2	1	3	3
	S: AM, E, MEM, STX	(0.0%)	(0.0%)	(0.0%)	(0.0%)	(0.0%)	(0.0%)	(0.0%)	(0.0%)	(0.0%)	(18.2%)	(9.1%)	(8.1%)	(2.5%)
**XII**	R: AM, E, MEM	0	1	0	1	1	0	1	2	0	0	0	0	3
	S: P, SXT	(0.0%)	(7.1%)	(0.0%)	(2.7%)	(3.7%)	(0.0%)	(14.3%)	(4.2%)	(0.0%)	(0.0%)	(0.0%)	(0.0%)	(2.5%)
**XIII**	R: P, E, MEM	1	0	0	1	1	0	1	2	0	0	0	0	3
	S: AM, SXT	(4.8%)	(0.0%)	(0.0%)	(2.7%)	(3.7%)	(0.0%)	(14.3%)	(4.2%)	(0.0%)	(0.0%)	(0.0%)	(0.0%)	(2.5%)
**XIV**	R: P, E, SXT	0	0	0	0	0	0	0	0	1	0	1	2	2
	S: AM, MEM	(0.0%)	(0.0%)	(0.0%)	(0.0%)	(0.0%)	(0.0%)	(0.0%)	(0.0%)	(7.7%)	(0.0%)	(9.1%)	(5.4%)	(1.7%)
**XV**	R: E	1	0	0	1	0	1	0	1	0	0	0	0	2
	S: P, AM, MEM, STX	(4.8%)	(0.0%)	(0.0%)	(2.7%)	(0.0%)	(7.1%)	(0.0%)	(2.1%)	(0.0%)	(0.0%)	(0.0%)	(0.0%)	(1.7%)
**XVI**	R: P, MEM	0	0	0	0	0	0	1	1	1	0	0	1	2
	S: AM, E, SXT	(0.0%)	(0.0%)	(0.0%)	(0.0%)	(0.0%)	(0.0%)	(14.3%)	(2.1%)	(7.7%)	(0.0%)	(0.0%)	(2.7%)	(1.7%)
**XVII**	R: P, AM, E, MEM	0	1	0	1	0	0	0	0	0	0	0	0	1
	S: SXT	(0.0%)	(7.1%)	(0.0%)	(2.7%)	(0.0%)	(0.0%)	(0.0%)	(0.0%)	(0.0%)	(0.0%)	(0.0%)	(0.0%)	(0.8%)
**XVIII**	R: P, AM, E	0	0	0	0	0	0	0	0	1	0	0	1	1
	S: MEM, SXT	(0.0%)	(0.0%)	(0.0%)	(0.0%)	(0.0%)	(0.0%)	(0.0%)	(0.0%)	(7.8%)	(0.0%)	(0.0%)	(2.7%)	(0.8%)
**XIX**	R: P, AM, E, SXT	0	0	0	0	0	0	0	0	0	1	0	1	1
	S: MEM	(0.0%)	(0.0%)	(0.0%)	(0.0%)	(0.0%)	(0.0%)	(0.0%)	(0.0%)	(0.0%)	(9.0%)	(0.0%)	(2.7%)	(0.8%)
**XX**	R: P, MEM, SXT	0	0	0	0	0	0	0	0	0	1	0	1	1
	S: AM, E	(0.0%)	(0.0%)	(0.0%)	(0.0%)	(0.0%)	(0.0%)	(0.0%)	(0.0%)	(0.0%)	(9.0%)	(0.0%)	(2.7%)	(0.8%)
**XXI**	R: P, AM, SXT	0	0	0	0	0	0	0	0	0	0	1	1	1
	S: MEM, E	(0.0%)	(0.0%)	(0.0%)	(0.0%)	(0.0%)	(0.0%)	(0.0%)	(0.0%)	(0.0%)	(0.0%)	(9.1%)	(2.7%)	(0.8%)
**XXII**	R: P, AM, MEM, SXT	0	0	0	0	1	0	0	1	0	0	0	0	1
	S: E	(0.0%)	(0.0%)	(0.0%)	(0.0%)	(3.7%)	(0.0%)	(0.0%)	(2.1%)	(0.0%)	(0.0%)	(0.0%)	(0.0%)	(0.8%)

P, penicillin; AM, ampicillin; MEM, meropenem; E, erythromycin; SXT, cotrimoxazole; -, lack of antibiotics.

## References

[1] Alía A., Andrade M.J., Córdoba J.J., Martín I., Rodríguez A. (2020). Development of a multiplex real-time PCR to differentiate the four major *Listeria monocytogenes* serotypes in isolates from meat processing plants. Food Microbiol..

[2] Liu Y., Sun W., Sun T., Gorris L.G.M., Wang X., Liu B., Dong Q. (2020). The prevalence of *Listeria monocytogenes* in meat products in China: A systematic literature review and novel meta-analysis approach. Int. J. Food Microbiol..

[3] Shamloo E., Hosseini H., Abdi Moghadam Z., Halberg Larsen M., Haslberger A., Alebouyeh M. (2019). Importance of *Listeria monocytogenes* in food safety: A review of its prevalence, detection, and antibiotic resistance. Iran. J. Vet. Res..

[4] Teixeira L.A.C., Carvalho F.T., Vallim D.C., Pereira R.C.L., Cunha Neto A., Vieira B.S., Carvalho R.C.T., Figueiredo E.E.S. (2019). *Listeria monocytogenes* in Export-approved Beef from Mato Grosso, Brazil: Prevalence, Molecular Characterization and Resistance to Antibiotics and Disinfectants. Microorganisms.

[5] European Food Safety Authority (2019). The European Union One Health 2018 Zoonoses Report. https://www.efsa.europa.eu/en/efsajournal/pub/5926.

[6] Braga V., Vázquez S., Vico V., Pastorino V., Mota M.I., Legnani M., Schelotto F., Lancibidad G., Varela G. (2017). Prevalence and serotype distribution of *Listeria monocytogenes* isolated from foods in Montevideo-Uruguay. Braz. J. Microbiol..

[7] Skowron K., Kwiecińska-Piróg J., Grudlewska K., Świeca A., Paluszak Z., Bauza-Kaszewska J., Wałecka-Zacharska E., Gospodarek-Komkowska E. (2018). The occurrence, transmission, virulence and antibiotic resistance of *Listeria monocytogenes* in fish processing plant. Int. J. Food Microbiol..

[8] Skowron K., Wiktorczyk N., Grudlewska K., Kwiecińska-Piróg J., Wałecka-Zacharska E., Paluszak Z., Gospodarek-Komkowska E. (2019). Drug-susceptibility, biofilm-forming ability and biofilm survival on stainless steel of *Listeria* spp. strains isolated from cheese. Int. J. Food Microbiol..

[9] RASFF (2018). The Rapid Alert System for Food and Feed, 2018 Annual Report. https://ec.europa.eu/food/sites/food/files/safety/docs/rasff_annual_report_2018.pdf.

[10] Carvalho F.T., Vieira B.S., Vallim D.C., Carvalho L.A., Carvalho R.C., Pereira R.C., Figueiredo E.E. (2019). Genetic similarity, antibiotic resistance and disinfectant susceptibility of *Listeria monocytogenes* isolated from chicken meat and chicken- meat processing environment in Mato Grosso, Brazil. LWT Food Sci. Technol..

[11] Filipello V., Mughini-Gras L., Gallina S., Vitale N., Mannelli A., Pontello M., Decastelli L., Allard M., Brown E.W., Lomonaco S. (2020). Attribution of *Listeria monocytogenes* human infections to food and animal sources in Northern Italy. Food Microbiol..

[12] Møller C.O., Sant’Ana A.S., Hansen S.K., Nauta M.J., Silva L.P., Alvarenga V.O., Maffei D., Silva F.F., Lopes J.T., Franco B.D. (2016). Evaluation of a cross contamination model describing transfer of *Salmonella* spp. and *Listeria monocytogenes* during grinding of pork and beef. J. Food Microbiol..

[13] Zhao Y., Teixeira J.S., Saldaña M.D.A., Gänzle M.G. (2019). Antimicrobial activity of bioactive starch packaging films against *Listeria monocytogenes* and reconstituted meat microbiota on ham. Int. J. Food Microbiol..

[14] Bolocan A.S., Nicolau A.I., Alvarez-Ordóñez A., Borda D., Oniciuc E.A., Stessl B., Gurgu L., Wagner M., Jordan K. (2016). Dynamics of *Listeria monocytogenes* colonisation in a newly-opened meat processing facility. Meat Sci..

[15] Desai A.N., Anyoha A., Madoff L.C., Lassmann B. (2019). Changing epidemiology of *Listeria monocytogenes* outbreaks, sporadic cases, and recalls globally: A review of ProMED reports from 1996 to 2018. Int. J. Infect. Dis..

[16] Smith A.M., Tau N.P., Smouse S.L., Allam M., Ismail A., Ramalwa N.R., Disenyeng B., Ngomane M., Thomas J. (2019). Outbreak of *Listeria monocytogenes* in South Africa, 2017–2018: Laboratory Activities and Experiences Associated with Whole-Genome Sequencing Analysis of Isolates. Foodborne Pathog. Dis..

[17] Stessl B., Szakmary-Bräendle K., Vorberg U., Schoder D., Wagner M. (2019). Temporal analysis of the *Listeria monocytogenes* population structure in floor drains during reconstruction and expansion of a meat processing plant. Int. J. Food Microbiol..

[18] European Food Safety Authority (2018). Multi-Country Outbreak of *Listeria monocytogenes* Serogroup IVb, Multi-Locus Sequence Type 6, Infections Linked to Frozen Corn and Possibly to Other Frozen Vegetables—First Update. https://www.efsa.europa.eu/en/supporting/pub/en-1448.

[19] Maung A.T., Mohammadi T.N., Nakashima S., Liu P., Masuda Y., Honjoh K., Miyamoto T. (2019). Antimicrobial resistance profiles of *Listeria monocytogenes* isolated from chicken meat in Fukuoka, Japan. Int. J. Food Microbiol..

[20] Baquero F., Lanza V., Duval M., Coque T.M. (2020). Ecogenetics of antibiotic resistance in *Listeria monocytogenes*. Mol. Microbiol..

[21] ISO, PNEN (2005). Microbiology of the Food Chain—Horizontal Method for the Detection and Enumeration of *Listeria monocytogenes* and of *Listeria* spp.—Part 1: Detection Method.

[22] Skowron K., Hulisz K., Gryń G., Olszewska H., Wiktorczyk N., Paluszak Z. (2018). Comparison of selected disinfectants efficiency against *Listeria monocytogenes* biofilm formed on various surfaces. Int. Microbiol..

[23] Centers for Disease Control and Prevention (2013). Standard Operating Procedure for PulseNet PFGE of Listeria monocytogenes.

[24] Doumith M., Buchrieser C., Glaser P., Jacquet C., Martin P. (2004). Differentiation of the major *Listeria monocytogenes* serovars by multiplex PCR. J. Clin. Microbiol..

[25] Wałecka-Zacharska E., Kosek-Paszkowska K., Bania J., Karpíšková R., Stefaniak T. (2012). Salt stress-induced invasiveness of major *Listeria monocytogenes* serotypes. Lett. Appl. Microbiol..

[26] EUCAST (2018). European Committee on Antimicrobial Susceptibility Testing (2018) Breakpoints Tables for Interpretation of MICs and Zones Diameters. Version 8.0.

[27] Pesavento G., Ducci B., Nieri D., Comodo N., Lo Nostro A. (2010). Prevalence and antibiotic susceptibility of *Listeria* spp. isolated from raw meat and retail foods. Food Control.

[28] Gonçalves-Tenório A., Silva B.N., Rodrigues V., Cadavez V., Gonzales-Barron U. (2018). Prevalence of Pathogens in Poultry Meat: A Meta-Analysis of European Published Surveys. Foods.

[29] Gamboa Marin Y., Buitrago S., Pérez-Pérez K., Marcela R., Poutou-Piñales R., Carrascal-Camacho A. (2012). Prevalence of *Listeria monocytogenes* in pork-meat and other processed products from the Colombian swine industry. Revista MVZ Córdoba.

[30] Wang K., Ye K., Zhu Y., Huang Y., Wang G., Wang H., Zhou G. (2015). Prevalence, antimicrobial resistance and genetic diversity of *Listeria monocytogenes* isolated from chilled pork in Nanjing, China. LWT Food Sci. Technol..

[31] Kuan C.H., Wong W.C., Pui C.F., Mahyudin N.A., Tang J.Y.H., Nishibuchi M., Radu S. (2014). Prevalence and quantification of *Listeria monocytogenes* in beef offal at retail level in Selangor, Malaysia. Braz. J. Microbiol..

[32] Komora N., Bruschi C., Magalhães R., Ferreira V., Teixeira P. (2017). Survival of *Listeria monocytogenes* with different antibiotic resistance patterns to food-associated stresses. Int. J. Food Microbiol..

[33] Mc Nulty K., Soon J.M., Wallace C.A., Nastasijevic I. (2016). Antimicrobial resistance monitoring and surveillance in the meat chain: A report from five countries in the European Union and European Economic Area. Trends Food Sci. Technol..

[34] Rakic-Martinez M., Drevets D.A., Dutta V., Katic V., Kathariou S. (2011). *Listeria monocytogenes* strains selected on ciprofloxacin or the disinfectant benzalkonium chloride exhibit reduced susceptibility to ciprofloxacin, gentamicin, benzalkonium chloride, and other toxic compounds. Appl. Environ. Microbiol..

[35] Oliveira T.S., Varjao L.M., da Silva L.N.N., de Castro Lisboa Pereira R., Hofer E., Vallim D.C., Almeida R.C.D.C. (2018). *Listeria monocytogenes* at chicken slaughterhouse: Occurrence, genetic relationship among isolates and evaluation of antimicrobial susceptibility. Food Control.

[36] Khen B.K., Lynch O.A., Carroll J., McDowell D.A., Duffy G. (2015). Occurrence, antibiotic resistance and molecular characterization of *Listeria monocytogenes* in the beef chain in the Republic of Ireland. Zoonoses Public Health.

[37] Gómez D., Azón E., Marco N., Carramiñana J.J., Rota C., Ariño A., Yangüela J. (2014). Antimicrobial resistance of *Listeria monocytogenes* and *Listeria innocua* from meat products and meat-processing environment. Food Microbiol..

[38] Jorgensen J., Waite-Cusic J., Kovacevic J. (2020). Prevalence of *Listeria* spp. in produce handling and processing facilities in the Pacific Northwest. Food Microbiol..

[39] Sereno M.J., Viana C., Pegoraro K., da Silva D.A.L., Yamatogi R.S., Nero L.A., Bersot L.D.S. (2019). Distribution, adhesion, virulence and antibiotic resistance of persistent *Listeria monocytogenes* in a pig slaughterhouse in Brazil. Food Microbiol..

[40] Kovacevic J., Sagert J., Wozniak A., Gilmour M.W., Allen K.J. (2013). Antimicrobial resistanceand co- selection phenomenon in *Listeria* spp. recovered from food and food production environments. Food Microbiol..

[41] Noll M., Kleta S., Al Dahouk S. (2017). Antibiotic susceptibility of 259 *Listeria monocytogenes* strains isolated from food, food-processing plants and human samples in Germany. J. Infect. Public Health.

[42] Barbuti S., Maggi A., Casoli C. (1992). Antibiotic resistance in strains of *Listeria* spp. from meat products. Lett. Appl. Microbiol..

[43] Rakic Martinez M., Wiedmann M., Ferguson M., Datta A.R. (2017). Assessment of *Listeria monocytogenes* virulence in the *Galleria mellonella* insect larvae model. PLoS ONE.

[44] Fox E., deLappe N., Garvey P., McKeown P., Cormican M., Leonard N., Jordan K. (2012). PFGE analysis of *Listeria monocytogenes* isolates of clinical, animal, food and environmental origin from Ireland. J. Med. Microbiol..

[45] Nadon C.A., Woodward D.L., Young C., Rodgers F.G., Wiedmann M. (2011). Correlations between Molecular Subtyping and Serotyping of *Listeria monocytogenes*. J. Clin. Microbiol..

[46] Chen J.Q., Regan P., Laksanalamai P., Healey S., Hu Z. (2017). Prevalence and methodologies for detection, characterization and subtyping of *Listeria monocytogenes* and *L. ivanovii* in foods and environmental sources. Food Sci. Hum. Wellness.

[47] Feng Y., Yao H., Chen S., Sun X., Yin Y., Jiao X. (2020). Rapid detection of hypervirulent serovar 4h *Listeria monocytogenes* by multiplex PCR. Front. Microbiol..

[48] Alía A., Andrade M.J., Rodríguez A., Martín I., Pérez-Baltar I., Medina M., Córdoba J.J. (2020). Prevalence and characterization of *Listeria monocytogenes* in deboning and slicing areas of Spanish dry-cured ham processing. LWT.

[49] Duranti A., Sabbatucci M., Blasi G., Acciari V., Ancora M., Bella A., Busani L., Centorame P., Cammà C., Conti F. (2018). A severe outbreak of listeriosis in central Italy with a rare pulsotype associated with processed pork products. J. Med. Microbiol..

[50] Lotfollahi L., Chaharbalesh A., Rezaee M.A., Hasani A. (2017). Prevalence, antimicrobial susceptibility and multiplex PCR-serotyping of *Listeria monocytogenes* isolated from humans, foods and livestock in Iran. Microb. Pathog..

